# Supplementary material for: Insect herbivory dampens Subarctic birch forest C sink response to warming
Source: Nat Commun. 2020 May 21;11:2529. doi: 10.1038/s41467-020-16404-4 (PMC7242322; doi:10.1038/s41467-020-16404-4)
Supplement: Supplementary file 1 — Supplementary Information [file 41467_2020_16404_MOESM1_ESM.pdf]

Supplementary Information for

Insect herbivory dampens Subarctic birch forest C sink response to warming

Silfver et al.

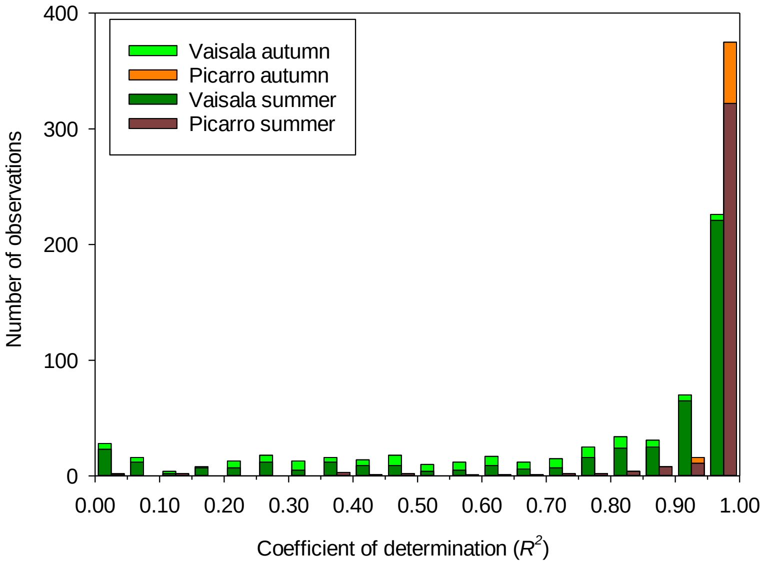

Supplementary Figure 1. The distribution of R^2 values of linear regressions used in the CO_2 flux calculation. Online, fast-response (~1 Hz) sampling of concentration changes in the chamber facilitates the calculation procedure due to a large number of data points, resulting in a small measurement error. The vast majority of R^2 values for the Picarro G2401-based measurements were >0.95. With the Vaisala GMP343-based system, the R^2 values were lower, especially with the low fluxes during autumn. However, the lower R^2 values were mostly due to the random analyzer noise and only 12 of the 1020 linear regressions fitted to the data had a $P > 0.05$ (with two-sided significance tests). All of these cases were related to a small CO_2 flux (–0.009 to 0.004 mg CO_2 m^{-2} s^{-1}). Source data are provided as a Source Data file.

a

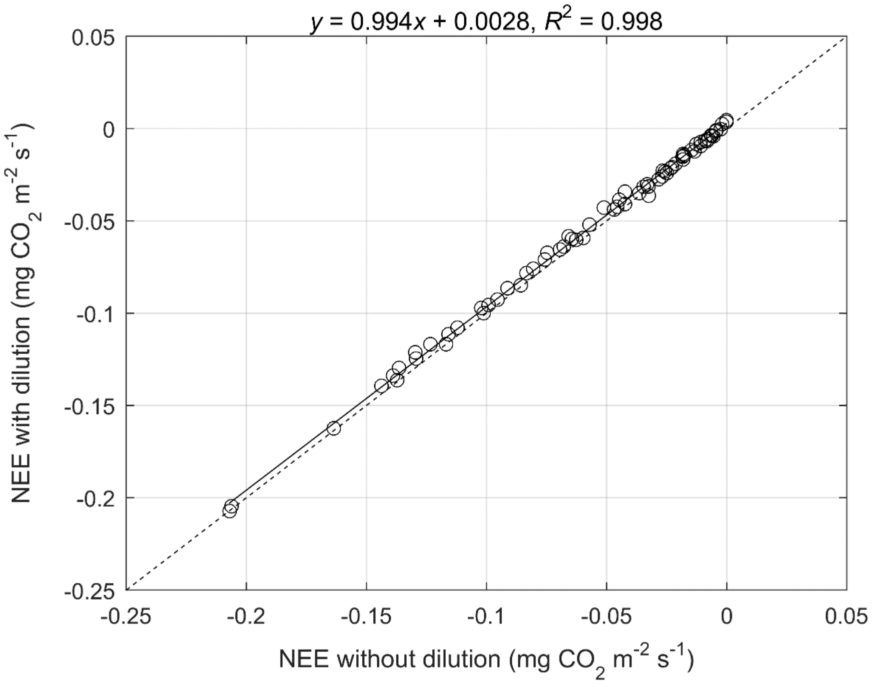

b

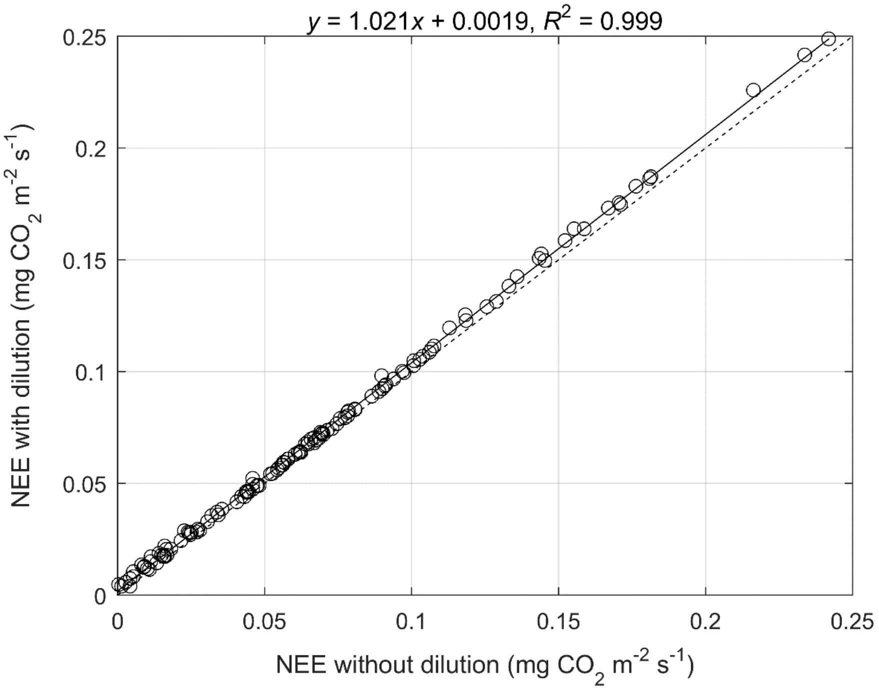

Supplementary Figure 2. Scatterplots of net ecosystem exchange NEE fluxes with and without dilution correction in the data recorded using the Vaisala GMP343 analyzer for **a** negative and **b** positive fluxes. The CO_2 mixing ratios measured using the Picarro G2401 are internally corrected for water vapour. In the GMP343-based system, we measured the relative humidity inside the chamber, but the sensor broke down during the measurement period. As shown here, the dilution effect is very limited and no correction was applied to these data. Source data are provided as a Source Data file.

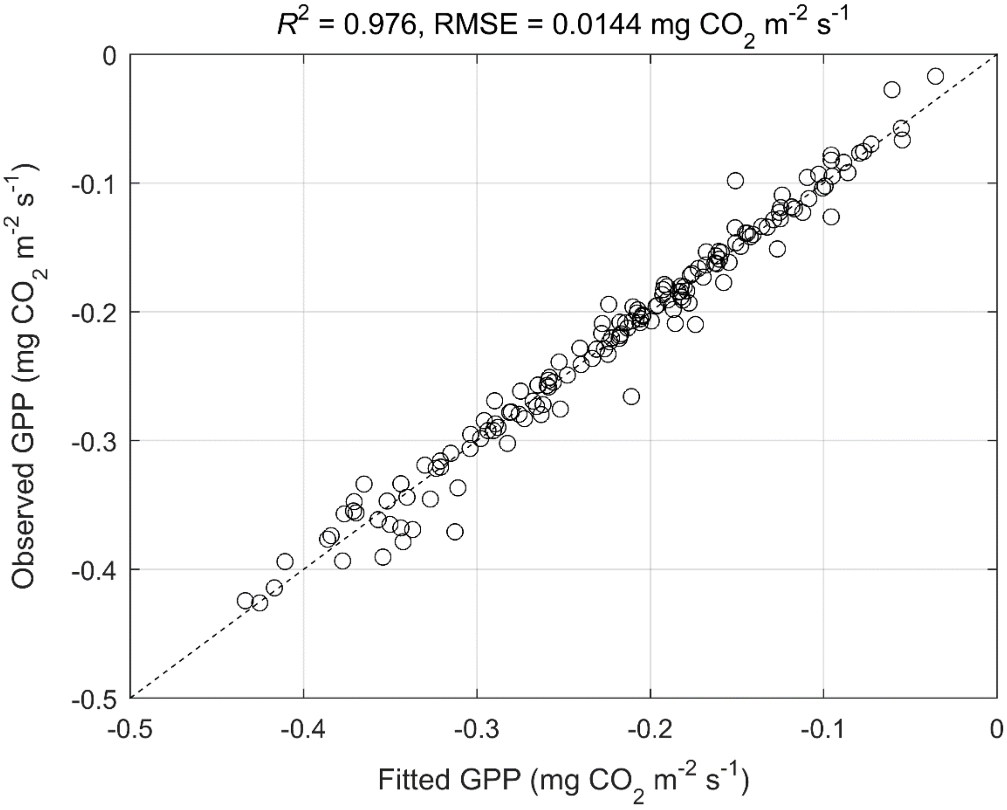

Supplementary Figure 3. Measured gross primary production GPP versus the GPP calculated using the fitted α and GP_{max} values. These values were used for determining the constant GP_{max}/α ratio, which was then used for standardizing the fluxes at PAR = 800 µmol m^{-2} s^{-1}. The ratio was determined as the median from those α and GP_{max} values estimated from the PAR response measurements that included three or four light levels and had the highest PAR > 800 µmol m^{-2} s^{-1}. The four-level cases are shown in the figure. Source data are provided as a Source Data file.

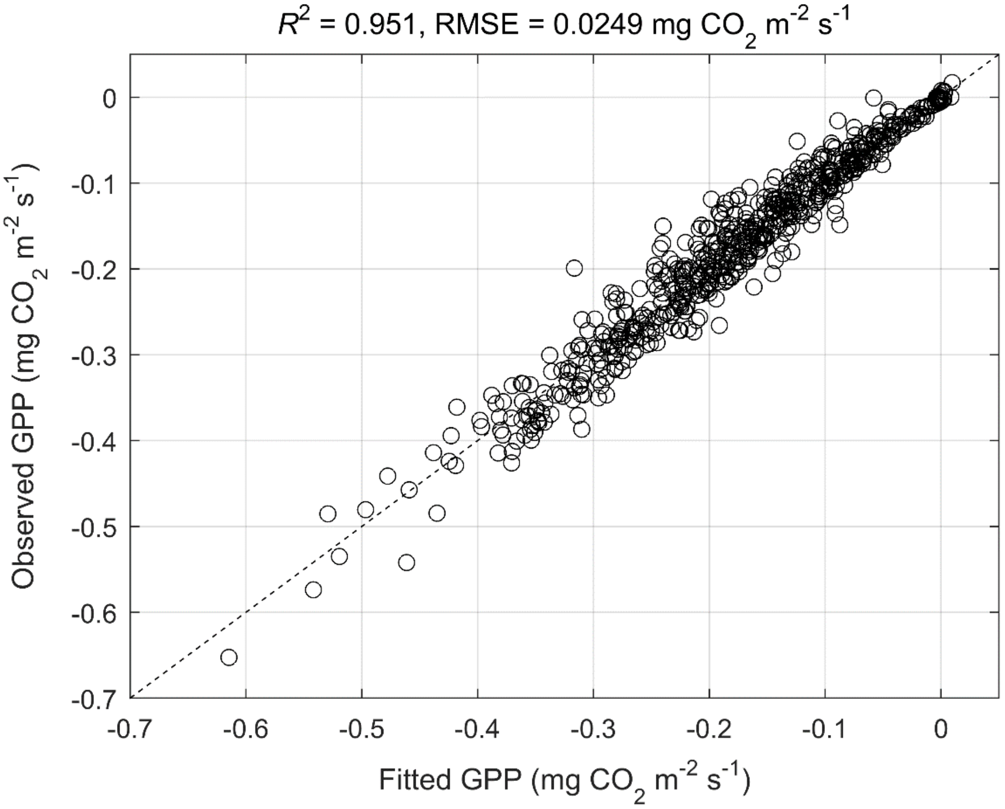

Supplementary Figure 4. The measured GPP versus the GPP calculated using the constant GP_{max}/α ratio (of 203 μmol m^{-2} s^{-1}) and the α parameter estimated for each PAR response measurement. The figure shows all cases in which three or four light levels were available. Source data are provided as a Source Data file.

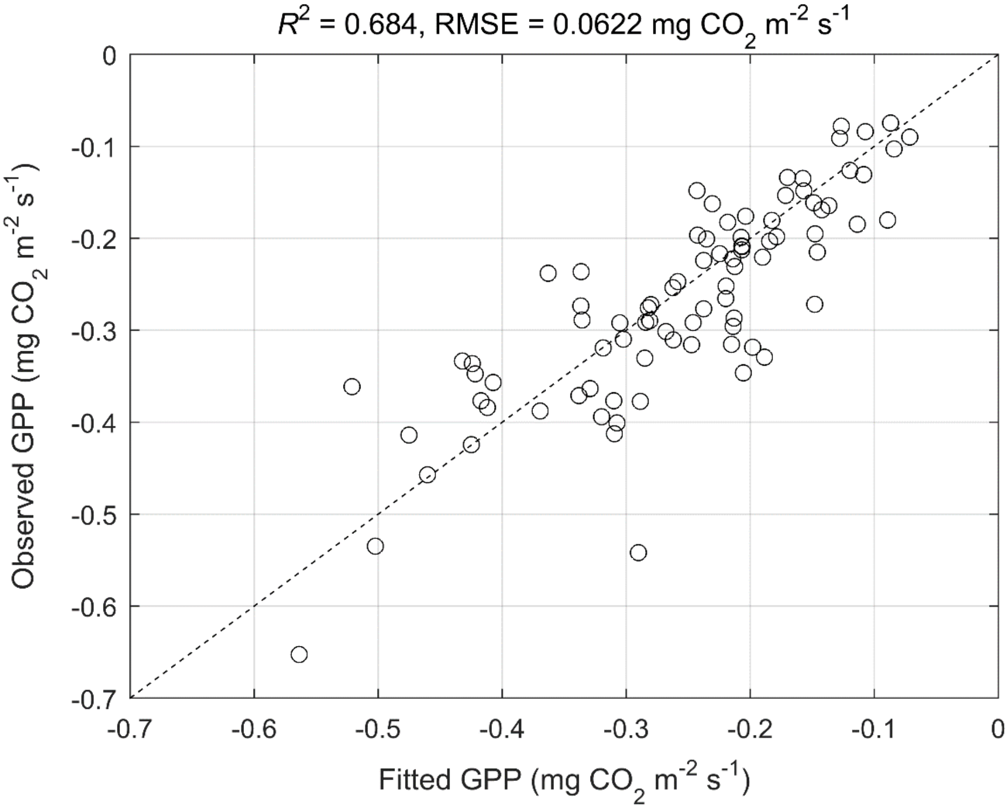

Supplementary Figure 5. Measured GPP versus the predicted GPP at the highest radiation level, based on a response function fit in which this data point was excluded when estimating the α parameter. This illustrates the uncertainty related to extrapolation of the PAR response beyond the highest light level observed. All data with at least three light levels and the highest PAR within 500–1100 µmol m^{-2} s^{-1} were included. Source data are provided as a Source Data file.

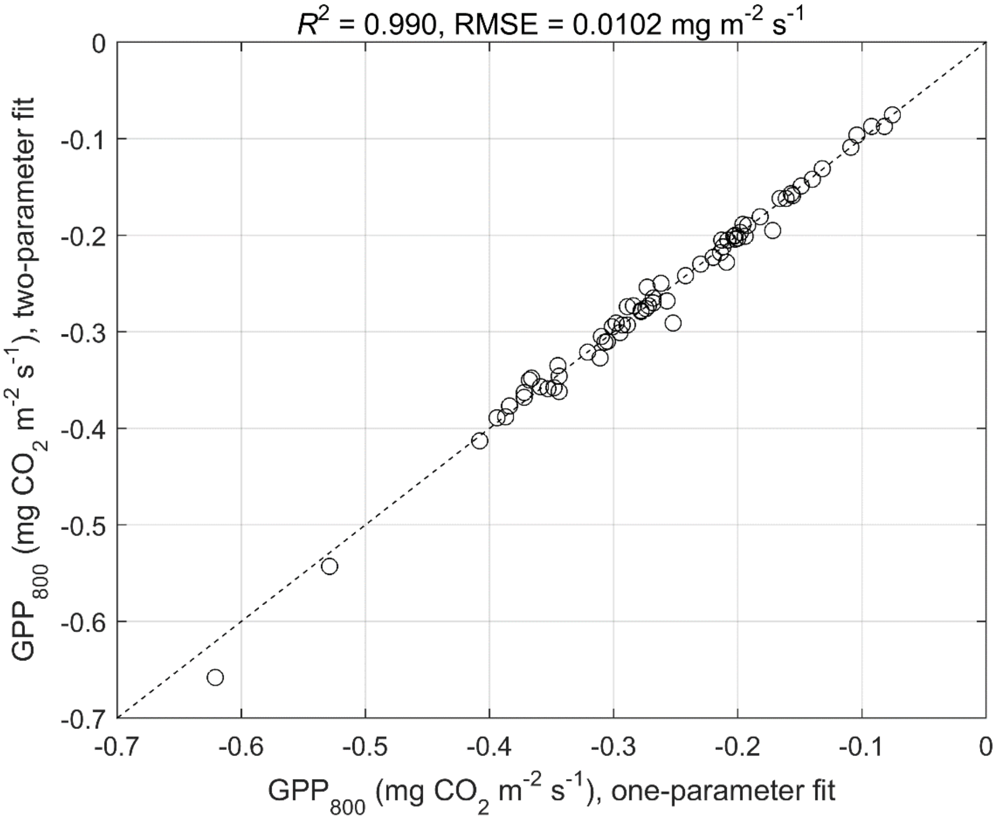

Supplementary Figure 6. GPP_{800} calculated with fits based on two free parameters (α and GP_{max}) versus the GPP_{800} calculated with fits based on one free parameter (α) and a fixed α/GP_{max} ratio. This illustrates the uncertainty related to using a constant α/GP_{max} in flux standardization. The data include the cases that were used for determining the fixed α/GP_{max}. Source data are provided as a Source Data file.

Supplementary Table 1. The mean percentage (s.e.m.) of leaves that belonged to the four damage categories (0, 1–4, 5–20 and > 20% of leaf area damaged) in *Betula* plantlets and the mean damage index (s.e.m.) calculated from these values in the four treatment combinations in 2017 (*n* = 57–60 per treatment) and 2019 (*n* = 44–55).

	0% damaged	1–4% damaged	5–20% damaged	> 20% damaged	Damage index
2017					
Control	74.3 (3.41)	22.9 (3.24)	2.2 (0.53)	0.5 (0.34)	0.38 (0.12)
Herbivory reduction (H)	89.1 (2.30)	10.5 (2.27)	0.3 (0.21)	0.1 (0.08)	0.07 (0.04)
Warming	76.6 (3.47)	18.9 (2.93)	2.9 (0.86)	1.7 (0.77)	0.38 (0.12)
Warming + H	87.0 (2.14))	10.4 (1.81)	2.4 (0.66)	0.1 (0.11)	0.18 (0.07)
2019					
Control	83.3 (2.76)	10.9 (2.30)	2.2 (0.63)	3.6 (1.04)	0.34 (0.11)
Herbivory reduction (H)	91.3 (1.51)	6.8 (1.12)	1.5 (0.72)	0.4 (0.31)	0.08 (0.04)
Warming	83.9 (2.91)	10.0 (1.70)	3.4 (0.89)	2.8 (1.09)	0.29 (0.10)
Warming + H	88.6 (1.66)	10.0 (1.48)	0.9 (0.41)	0.5 (0.40)	0.18 (0.07)

Source data are provided as a Source Data file.

Supplementary Table 2. Statistics of warming (ambient and +3 °C) and herbivory (normal and reduced) effects on leaf damage index in the additional 2019 survey (*N* = 193).

	F	*P*
Soil OM content	0.7	0.392
Vascular plant cover	13.6	**<0.001**
Lichen cover	0.4	0.509
Moss cover	0.5	0.494
Warming (W)	0.8	0.376
Herbivory reduction (H)	16.4	**<0.001**
Betula species (S)	5.6	**0.022**
W×H	3.1	0.078
W×S	0.7	0.577
H×S	3.0	**0.034**
W×H×S	0.2	0.922

The data was log-transformed and analyzed using linear mixed models and Type I Anova (with two-sided significance tests). Soil OM content and the areal cover of vascular plants, mosses and lichens were treated as covariates and added to the model to remove plot-to-plot variation that might otherwise confound the treatment effects. Field replicate block and birch genotype (nested within species) were included in the model as random effects, but are not reported. Values of *P* < 0.05 are in bold.

Supplementary Table 3. Statistics of warming (ambient and +3 °C) and herbivory (normal and reduced) effects on bud break timing and the relative growth of the experimental birch plantlets in 2017 and 2018 (*N* = 232–239 for each year).

	Bud break timing		Relative growth	
	F	*P*	*F*	*P*
Soil OM content	0.6	0.434		
Vascular plant cover	9.0	**0.003**		
Lichen cover	0.4	0.560	5.2	**0.023**
Moss cover	40.0	**<0.001**		
Year (Y)	4835	**<0.001**	225.1	**<0.001**
Warming (W)	39.5	**<0.001**	4.0	**0.046**
Herbivory reduction (H)	15.8	**<0.001**	10.4	**<0.001**
Betula species (S)	1.5	0.283	0.1	0.940
W×Y	11.1	**<0.001**	2.5	0.113
H×Y	3.0	0.083	10.1	**0.002**
S×Y	8.3	**<0.001**	6.0	**0.001**
W×H	1.8	0.185	0.6	0.434
W×S	7.0	**<0.001**	0.1	0.964
H×S	1.2	0.319	1.2	0.293
W×H×Y	2.8	0.095	0.0	0.955
W×S×Y	5.3	**0.001**	0.4	0.781
H×S×Y	1.4	0.254	0.9	0.439
W×H×S	0.1	0.955	1.6	0.201
W×H×S×Y	0.4	0.734	0.3	0.847

The data were analyzed using repeated measures linear mixed models and Type I Anova (with two-sided significance tests). Year was treated in the models as a repeated measure. Soil OM content and the areal cover of vascular plants, mosses and lichens were treated as covariates and added to models to remove plot-to-plot variation that might otherwise confound the treatment effects (but omitted from the final model if redundant). Field replicate block and birch genotype (nested within species) were included in the models as random effects, but are not reported. Relative growth was log-transformed before analysis. Values of $P < 0.05$ are in bold.

Supplementary Table 4. Statistics of warming (ambient and +3 °C) and herbivory (normal and reduced) effects on the summer and autumn leaf chlorophyll content of the experimental birch plantlets in 2017–2018.

	Summer 2017		Summer 2018		Autumn 2017		Autumn 2018	
	F	*P*	*F*	*P*	*F*	*P*	*F*	*P*
Soil OM content	0.3	0.600	0.9	0.354	2.2	0.142	0.5	0.506
Vascular plant cover	1.1	0.290	10.5	**0.001**	1.2	0.267	9.0	**0.003**
Lichen cover	0.0	0.965	0.0	0.834	2.3	0.129	8.3	**0.005**
Moss cover	16.4	**<0.001**	50.8	**<0.001**	42.7	**<0.001**	70.8	**<0.001**
Date (D)	14.1	**<0.001**	575.7	**<0.001**	657.4	**<0.001**	602.0	**<0.001**
Warming (W)	5.1	**0.025**	19.7	**<0.001**	33.4	**<0.001**	83.3	**<0.001**
Herbivory reduction (H)	13.3	**<0.001**	26.5	**<0.001**	10.9	**0.001**	24.1	**<0.001**
Betula species (S)	3.2	0.083	1.6	0.274	12.2	**0.003**	17.5	**0.001**
W×D	3.3	0.071	6.8	**0.001**	15.1	**<0.001**	18.7	**<0.001**
H×D	1.9	0.173	7.5	**0.001**	2.3	**0.010**	1.5	0.131
S×D	3.8	**0.011**	1.8	0.100	19.6	**<0.001**	11.1	**<0.001**
W×H	0.5	0.465	4.1	**0.043**	0.0	0.903	6.2	**0.014**
W×S	0.2	0.868	0.8	0.506	8.2	**<0.001**	13.8	**<0.001**
H×S	0.5	0.717	2.4	0.066	1.4	0.246	2.4	0.073
W×H×D	0.4	0.549	2.1	0.129	2.0	**0.026**	0.6	0.812
W×S×D	0.8	0.490	0.6	0.736	6.2	**<0.001**	14.7	**<0.001**
H×S×D	0.3	0.803	1.0	0.455	0.6	0.975	0.9	0.617
W×H×S	0.1	0.979	1.5	0.213	0.3	0.834	1.5	0.227
W×H×S×D	1.4	0.256	0.6	0.700	0.5	0.986	0.7	0.907

The data were analyzed using repeated linear mixed models and Type I Anova (with two-sided significance tests). Date was treated in the models as a repeated measure (2–3 dates for summers, 12–10 dates for autumns, N = 234–235 for 2017, N = 181–235 for 2018). Soil OM content and the areal cover of vascular plants, mosses and lichens were treated as covariates and added to models to remove plot-to-plot variation that might otherwise confound the treatment effects. Field replicate block and birch genotype (nested within species) were included in the models as random effects, but are not reported. Values of $P < 0.05$ are in bold.

Supplementary Table 5. Statistics of warming (ambient and +3 °C) and herbivory (normal and reduced) effects on (a) the resin capture of soil mineral N (sum of NH_4^+ and NO_3^-) and (b) soil microbial biomass carbon (MBC).

**(a)** Mineral N	*F*	*P*
Soil OM content	3.0	0.116
Vascular plant cover	4.5	0.065
Lichen cover	21.1	**0.001**
Moss cover	5.6	**0.041**
Year (Y)	5.6	**0.030**
Warming (W)	2.3	0.167
Herbivory reduction (H)	12.1	**0.008**
W×H	5.3	**0.050**
W×Y	0.04	0.849
H×Y	1.2	0.285
W×H×Y	0.03	0.872

**(b)** MBC	*F*	*P*
Soil OM content	0.9	0.370
Vascular plant cover	5.3	**0.045**
Lichen cover	4.9	**0.049**
Moss cover	1.4	0.259
Soil layer (L)	3951	**<0.001**
Warming (W)	0.2	0.649
Herbivory reduction (H)	3.8	0.085
W×H	12.0	**0.008**
W×L	1.2	0.325
H×L	4.4	**0.020**
W×H×L	0.3	0.772

Data were analyzed using repeated measures linear mixed models and Type I Anova (with two-sided significance tests). Year (mineral N data, $n = 20$ for each year) and soil layer (three layers, MBC data, $n = 20$ for each layer) were treated in the models as repeated measures. Soil OM content and the areal cover of vascular plants, mosses and lichens were treated as covariates and added to models to remove plot-to-plot variation that might otherwise confound the treatment effects (but omitted from the final model if redundant). Field replicate block was included in the models as a random effect, but is not reported. Mineral N was log-transformed and MBC square-root transformed before analysis. Values of $P < 0.05$ are in bold.

Supplementary Table 6. Monthly mean air temperature and precipitation during the May–November warming period in the study years 2017 and 2018 and in the reference years 1981–2010.

	Mean temperature (°C)			Precipitation (mm)		
	2017	2018	1981–2010	2017	2018	1981–2010
May	1.9	7.2	3.7	11	13	19
June	7.8	9.4	9.6	49	52	42
July	13.6	18.1	13.1	161	56	62
August	10.4	11.4	10.7	63	111	48
September	6.5	7.1	5.7	34	70	30
October	0.7	-1.2	-0.5	12	15	33
November	-8.2	-1.7	-8.3	42	23	24

Data from Kevo weather station (Finnish Meteorological Institute), ca. 200 m from the study site.

Supplementary Table 7. Statistics of warming (ambient and +3 °C) and herbivory (normal and reduced) on soil moisture in 2017 and 2018.

	2017		2018	
	F	*P*	*F*	*P*
Soil OM	1.0	0.337	0.1	0.325
Vascular plants	3.9	0.071	69.1	**0.001**
Lichens	1.1	0.321	12.0	**0.037**
Mosses	1.8	0.204	4.1	0.282
Date (D)	57.1	**<0.001**	166	**<0.001**
Warming (W)	1.6	0.303	4.4	0.272
Herbivory reduction (H)	0.0	0.969	0.0	0.674
D×W	0.9	0.440	4.2	0.499
D×H	0.2	0.878	0.5	0.982
W×H	0.0	0.993	1.8	0.500
W×H×D	0.3	0.816	4.4	0.054

The data were analyzed using repeated measures linear mixed models and Type I Anova (with two-sided significance tests). Date was treated in the models as a repeated measure (four and six dates for 2017 and 2018 respectively, $n = 20$ for each date). Soil OM content and the areal cover of vascular plants, mosses and lichens were treated as covariates and added to models to remove plot-to-plot variation that might otherwise confound the treatment effects. Field replicate block was included in the models as a random effect, but is not reported. Values of $P < 0.05$ are in bold.

Supplementary Table 8. Statistics of herbivory (normal and reduced) and warming (ambient and +3 °C) effects on net ecosystem CO_2 exchange (NEE) using different PAR levels for standardizing the fluxes.

	NEE_{1200}		NEE_{800}		NEE_{300}	
	F	*P*	*F*	*P*	*F*	*P*
2017						
Soil OM content	0.3	0.585	0.2	0.687	0.1	0.764
Vascular plant cover	10.4	**0.011**	9.3	**0.014**	**4.8**	**0.057**
Lichen cover	19.7	**0.001**	20.5	**0.001**	**24.3**	**0.001**
Moss cover	1.6	0.230	1.8	0.211	2.6	0.140
Date (D)	51.5	**<0.001**	46.5	**<0.001**	**27.6**	**<0.001**
Warming (W)	2.2	0.175	2.0	0.190	1.3	0.277
Herbivory reduction (H)	6.1	**0.037**	5.7	**0.043**	3.7	0.091
W×H	1.1	0.323	1.0	0.352	0.4	0.525
W×D	2.8	**0.034**	2.9	**0.029**	**3.3**	**0.016**
H×D	1.2	0.337	1.1	0.355	0.9	0.458
W×H×D	0.3	0.907	0.2	0.916	0.2	0.957
2018						
Soil OM content	0.1	0.811	0.02	0.895	0.2	0.680
Vascular plant cover	1.0	0.333	0.7	0.411	0.0	0.967
Lichen cover	7.8	**0.018**	8.2	**0.016**	10.3	**0.009**
Moss cover	5.4	**0.042**	5.2	**0.046**	4.2	0.070
Date (D)	32.7	**<0.001**	31.5	**<0.001**	28.0	**<0.001**
Warming (W)	13.3	**0.006**	13.2	**0.006**	12.2	**0.008**
Herbivory reduction (H)	8.7	**0.018**	8.3	**0.020**	6.2	**0.038**
W×H	0.5	0.507	0.4	0.534	0.2	0.687
W×D	1.5	0.142	1.5	0.129	1.7	0.076
H×D	1.3	0.208	1.3	0.215	1.2	0.269
W×H×D	1.5	0.149	1.5	0.154	1.4	0.191

Treatment effects on net ecosystem exchange (NEE_{1200}, NEE_{800}, NEE_{300}) were tested using repeated measures linear mixed models and Type I ANOVA (with two-sided significance tests), where the variance is allocated to explanatory variables in the order of their appearance. Soil organic matter (OM) content and cover of vascular plants, lichens and mosses are continuous variables that describe the variation among the experimental plots prior to the establishment of the experiment. They were used in the models as covariates to remove plot-to-plot variation that might otherwise confound the treatment effects. Years were analyzed separately (N = 100 for 2017, N = 240 for 2018). Date was treated as a repeated measure, warming and herbivory reduction as fixed effects and treatment block (not reported) as a random effect. *F* and *P* indicate *F*-statistics and *P*-values respectively; $P < 0.05$ are in bold. As GPP_{1200} and GPP_{300} are proportional to GPP_{800}, they produce the same results as GPP_{800} (Table 2). Values of $P < 0.05$ are in bold. Source data are provided as a Source Data file.
