# Supplementary material for: Insect herbivory dampens Subarctic birch forest C sink response to warming
Source: Nat Commun. 2020 May 21;11:2529. doi: 10.1038/s41467-020-16404-4 (PMC7242322; doi:10.1038/s41467-020-16404-4)
Supplement: Supplementary file 3 — Reporting Summary [file 41467_2020_16404_MOESM3_ESM.pdf]

nature research

Corresponding author(s): Silfver Tarja, 9.4.2020

Reporting Summary

Nature Research wishes to improve the reproducibility of the work that we publish. This form provides structure for consistency and transparency in reporting. For further information on Nature Research policies, see Authors & Referees and the Editorial Policy Checklist.

Statistical parameters

When statistical analyses are reported, confirm that the following items are present in the relevant location (e.g. figure legend, table legend, main text, or Methods section).

n/a	Confirmed	
☐	☒	The exact sample size (*n*) for each experimental group/condition, given as a discrete number and unit of measurement
☐	☒	An indication of whether measurements were taken from distinct samples or whether the same sample was measured repeatedly
☐	☒	The statistical test(s) used AND whether they are one- or two-sided<br>*Only common tests should be described solely by name; describe more complex techniques in the Methods section.*
☐	☒	A description of all covariates tested
☐	☒	A description of any assumptions or corrections, such as tests of normality and adjustment for multiple comparisons
☐	☒	A full description of the statistics including central tendency (e.g. means) or other basic estimates (e.g. regression coefficient) AND variation (e.g. standard deviation) or associated estimates of uncertainty (e.g. confidence intervals)
☐	☒	For null hypothesis testing, the test statistic (e.g. *F*, *t*, *r*) with confidence intervals, effect sizes, degrees of freedom and *P* value noted<br>*Give P values as exact values whenever suitable.*
☒	☐	For Bayesian analysis, information on the choice of priors and Markov chain Monte Carlo settings
☒	☐	For hierarchical and complex designs, identification of the appropriate level for tests and full reporting of outcomes
☒	☐	Estimates of effect sizes (e.g. Cohen's *d*, Pearson's *r*), indicating how they were calculated
☐	☒	Clearly defined error bars<br>*State explicitly what error bars represent (e.g. SD, SE, CI)*

Our web collection on statistics for biologists may be useful.

Software and code

Policy information about availability of computer code

Data collection	No software was used in data collection.
Data analysis	SPSS 21 was used for statistical analysis.

For manuscripts utilizing custom algorithms or software that are central to the research but not yet described in published literature, software must be made available to editors/reviewers upon request. We strongly encourage code deposition in a community repository (e.g. GitHub). See the Nature Research guidelines for submitting code & software for further information.

Data

Policy information about availability of data

All manuscripts must include a data availability statement. This statement should provide the following information, where applicable:

- Accession codes, unique identifiers, or web links for publicly available datasets
- A list of figures that have associated raw data
- A description of any restrictions on data availability

Data availability: The authors declare that the data supporting the findings of this study are available within the paper and its supplementary information files. Source Data for Figs. 1–3, Supplementary Figs. 1–6, Supplementary Tables 2 and 8 are provided with the paper.

Field-specific reporting

Please select the best fit for your research. If you are not sure, read the appropriate sections before making your selection.

☐ Life sciences ☐ Behavioural & social sciences ☒ Ecological, evolutionary & environmental sciences

For a reference copy of the document with all sections, see nature.com/authors/policies/ReportingSummary-flat.pdf

Ecological, evolutionary & environmental sciences study design

All studies must disclose on these points even when the disclosure is negative.

Study description	A field site in Subarctic mountain birch forest with altogether 20 treatment plots. The treatments included warming and herbivore reduction in a 2×2 factorial set-up with 5 replicates. When established, a total of 240 experimental birch plantlets were planted in treatment plots within intact ground layer vegetation (three genotypes from each of the four birch species, or twelve plantlets, in each plot).
Research sample	A total of 20 treatment plots with 240 planted birch saplings. The included birch species were Betula nana, B. pubescens subsp. czerepanovii, B. pendula and B. pubescens. Three genotypes were used for each species to get a representative estimate of each species' responses. Birch genotypes were cloned from individuals randomly chosen from Subarctic birch populations.
Sampling strategy	CO_2 exchange was measured in all treatment plots 17 times over the two growing seasons. All birch plantlets were observed for phenology, leaf chlorophyll content and growth across the two growing seasons, and for leaf herbivore damage during the first full growing season. Soil N availability was quantified in each plot for each year and the microbial biomass carbon in three soil layers of each plot in the second full growing season. No sample-size calculation was performed to determine the number of replicate plots in the field site, the chosen number was based on earlier experience in field plant and soil research. Five replicate plots is sufficient for statistical analyses when combined with a careful analysis of soil and vegetation covariates in each plot in the beginning of the experiment.
Data collection	Several researchers used the closed chamber technique to measure CO_2 flow under standard protocols. Birch phenology (bud break and leaf chlorophyll decline), growth and leaf damage were recorded by K.M. and T.S. Several researchers measured soil moisture and temperature using standard protocols. Air temperature data was collected using automatic sensors. Soil cores for microbial biomass carbon were collected and analyzed by N.M. and K.K.
Timing and spatial scale	CO_2 flow measurements (every 2 or 3 weeks) and phenological observations (bud break daily, autumn leaf chlorophyll concentrations twice a week) were carried out from early to late growing season across two years in all treatment plots of the field site. Data collection was started with bud break observations on 6th of June in 2017 and 14th of May in 2018 and stopped with the last chlorophyll measurement on 29th of September in 2017 and with the last CO_2 flux measurement on 22nd of October in 2018. Measurements of abiotic attributes, except for air temperature which was followed daily, were carried out every 2 or 3 weeks during mid summer. Based on earlier experience, these intervals were considered sufficient for observing treatment effects on the chosen response variables. The size of the experimental field site was circa 28 m x 9 m. The distance between adjacent treatment plots varied, but was always > 1.2 m.
Data exclusions	No data were excluded.
Reproducibility	Statistical analyses showed consistent results across the two study years. The data consist of observations collected from one experiment at one field site, the experiment was not replicated in several sites.
Randomization	Randomized block design was used for the field site i.e. the four treatment combinations were placed in random locations within each of the five field blocks. Birch saplings were randomly allocated to planting spots within each treatment plot.
Blinding	Blinding was not possible during field measurements and data collection as treatments were clearly visible in the field. Laboratory analysis of soil N and microbial biomass carbon were blinded.

Did the study involve field work? ☒ Yes ☐ No

Field work, collection and transport

Field conditions	In the field site location, the long-term (1981-2010) mean temperatures for May, June, July, August and September are 3.7, 9.6, 13.1, 10.7 and 5.7 °C and the mean precipitation is 19, 42, 62, 48 and 30 mm, respectively. In 2017, monthly mean temperatures followed the long-term means, while July and August precipitation was higher. In 2018, May and July were significantly warmer than the long-term means, while precipitation for these months followed the long-term means and was higher than on average later in August and September. In July 2018, the daily maximum atmospheric water vapor pressure deficit (VPD) was frequently above 2.5 kPa, indicating severe moisture stress for this month.
Location	Utsjoki, Kevo, northern Finland (69°45′N, 27°01′E)
Access and import/export	The field site and the experiment are situated in the premises of the Turku University Kevo Subarctic Research Institute. Birch plantlets were produced and imported to the site from the Haapastensyrjä Unit of the Natural Resources Institute Finland (Luke),

situated in southern Finland, while the soil samples and the resin capsules collected at the experimental site were exported and analysed at the University of Helsinki laboratories in Helsinki and Lahti. No permits were required for such national transfer of plant material and samples.

Disturbance	Vegetation in the space between treatment plots got disturbed by trampling during the experiment. Treatment plots were surrounded with a line and no disturbance was allowed on the plots.

Reporting for specific materials, systems and methods

Materials & experimental systems

n/a	Involved in the study
☒	☐ Unique biological materials
☒	☐ Antibodies
☒	☐ Eukaryotic cell lines
☒	☐ Palaeontology
☒	☐ Animals and other organisms
☒	☐ Human research participants

Methods

n/a	Involved in the study
☒	☐ ChIP-seq
☒	☐ Flow cytometry
☒	☐ MRI-based neuroimaging
